# Group B Streptococcus colonization rate and serotype distribution among pregnant women and their newborns at Adama Hospital Medical College, Ethiopia

**DOI:** 10.1038/s41598-020-66474-z

**Published:** 2020-06-09

**Authors:** Musa Mohammed Ali, Daniel Asrat, Demissie Assegu Fenta, Tolossa Eticha Chaka, Yimtubezinash Woldeamanuel

**Affiliations:** 1 Hawassa University College of Medicine and Health Sciences, Hwassa, Ethiopia; 2 Addis Ababa University College of Health Science, Department of Microbiology, Immunology and Parasitology, Addis Ababa, Ethiopia; 3 Adama Hospital Medical College, Adama, Ethiopia

**Keywords:** Infectious diseases, Microbiology

## Abstract

Rectovaginal area of pregnant women can be colonized transiently with group B Streptococcus (GBS) without causing disease. The bacteria can be transmitted to the newborn before and during birth and cause early-onset neonatal disease. In this study, we aimed to determine the GBS colonization rate among pregnant women before delivery and their newborns and serotypes distribution of GBS. Two hundred-eighty pregnant women along with their newborns were screened for GBS colonization from June 2014 to October 2014 at Adama Hospital Medical College. Rectovaginal swabs from pregnant women before delivery and specimen from nasal area, external ear, umbilical cord and throat of newborns were collected and cultured. The serotyping of GBS was performed by using serotype-specific antisera. To collect sociodemographic and clinical data we employed a structured questionnaire. GBS colonization among pregnant women and their newborns were 13.2% 95% CI (8.9–17.5) and 7.4% 95% CI (4.6–10.6). Out of 37 GBS strains recovered from pregnant women, the prevalent serotypes were Ia 6(16.2%), Ib 8(21.6%), II 10(27%), III 3(8.1%), and V 8(21.6%). Out of 21 GBS strains recovered from newborns, prevalent serotypes were Ia 3(14.3%), Ib 6(28.6%), II 6(28.6%), III 4(19%), and V 1(4.8%). This study indicated the existence of primary risk factors for neonatal disease in Adama area. Serotype II was the common serotype detected in this study which is followed by serotype Ib, Ia, and V. As colonizing GBS serotypes could cause invasive disease among newborns, vaccine formulation which includes serotype II, Ia, V, Ib, and III can prevent of invasive disease caused by GBS in the study area.

## Introduction

In 1970 GBS emerged as a the main cause of neonatal morbidity and mortality in the United States of America (USA) and other industrialized countries^1,2^. Neonatal GBS disease can be classified as early-onset disease (EOD), which occurs in less than 7 days after birth and late onset-disease (LOD) which occurs in between 7 and 90 days after birth^3^.

The primary risk factor for EOD is rectovaginal colonization of pregnant women with GBS during delivery^4^. The maternal GBS colonization rate varies in different settings. The lowest colonization rate was reported from India, 7.6%^5^ and the highest was reported from Norway, 34.8%^6^. Factors such as prolonged rupture of membrane, prematurity, chorioamnionitis, low level of anti-GBS capsular antibody and previous newborn with EOD can increase the risk of disease among newborns^7,8^. Newborns can acquire the disease due to vertical transmission of GBS from the colonized mother to their newborns *in utero* or during passage through birth canal. The transmission mechanism for LOD is not well known^4,8^.

Administration of Intrapartum Antibiotic Prophylaxis (IAP) for GBS colonized pregnant women before delivery or for pregnant women with risk factors can reduce EOD due to GBS. The prevention strategy, IAP, was issued in 1996 by a different organization and professional association in the USA. The strategy when first released, it reduced a significant amount of EOD caused by GBS. The strategy was updated in 2002 and 2010 to further reduce neonatal disease caused by GBS^1^.

Even though IAP has substantially reduced EOD caused by GBS, it has several limitations. The strategy does not eliminate all cases of EOD; it does not affect LOD caused by GBS and there is a concern of the selection of antimicrobial resistance bacteria^3^. Use of IAP has reduced about 80% burden of EOD due to GBS, out of 1.8 newborns per 1000 live births in the 1990s to 0.23 newborns per 1000 live births in 2015^9^.

Above all, screening based IAP is not feasible for developing countries where resource is limited for laboratory diagnosis. As an alternative, the capsular polysaccharide based vaccine is being developed; currently, vaccine formulation which contains GBS serotype such as Ia, Ib, and III has completed phase II clinical trial and it was reported to be cost-effective^10^. However as GBS serotypes vary from place to place and from time to time the current vaccine formulation may not work equally for all countries^6–8^. As a result data on epidemiology of GBS serotype is required from every country. In Ethiopia, there is scarce data on maternal GBS colonization and GBS serotype distribution. Therefore, this study was sought to provide valuable data on maternal and newborns GBS colonization rate, associated risk factors and serotypes distribution.

## Methods

### Study area

Adama Hospital Medical College (AHMC) is located at Adama City, Oromiya regional state; it is located 100 km due east of Addis Ababa. The City has a total population of 220,212. It is located at 8°33′N39°16′E/8.55°N39.27°E at an elevation of 1712 meters.

### Study design

A Hospital-based cross-sectional study was conducted from June 2014-October 2014 at Adama Hospital Medical College, Adama, Ethiopia.

### Study population

Out of pregnant women who were admitted at Adama Hospital Medical College for delivery during the study period, 280 along with their newborns were consented and screened for GBS colonization. Two hundred eighty pregnant women who fulfilled the inclusion criteria were recruited based on a convenience sampling technique. Neonates born from GBS colonized mother were followed through telephone for 7 days. The sample size was calculated by using a single proportion formula, margin of error = 0.05, Confidence Interval = 95%, and prevalence from previous study conducted in Ethiopia, 20.86%^11^.

### Inclusion and exclusion criteria

Pregnant women with normal delivery were included. Pregnant women who were on antibiotic for the last three weeks and cesarean-section delivery were excluded.

### Study variables

#### Dependent variable

Maternal and newborn GBS colonization rate, Serotype distribution

#### Independent variables

Associated factors

### Data collection

#### Sample collection, handling, and transport

Rectovaginal specimens were collected from pregnant women and the specimen was collected from nasal area, external ear, umbilical cord and throat of the newborns by the trained midwifery from informed and consented pregnant women before delivery and was placed in labeled Stuart’s transport media (BD Diagnostics, USA). The specimens were transported to the Microbiology Laboratory within 4 hours after collection. Socio-demographic and clinical data were collected by using a structured questionnaire^2,12^.

### Identification and serotyping of group B Streptococci

All specimens collected from pregnant women and newborns were placed in selective media, Lim broth (BD Diagnostics, USA). After inoculation, the media was incubated for 18–24 hours, at 37 °C in CO_2_ atmosphere. On the next day, it was sub-cultured onto sheep blood agar plate (BD Diagnostics, USA) and incubated in the CO_2_ enriched atmosphere for 18 − 24 hours. If GBS was not detected, the blood agar plate was re-incubated and examined after 48 hours to detect GBS. All colonies which were beta-hemolytic, or non-hemolytic, Gram positive cocci, catalase-negative was sub-cultured and isolated for confirmatory testing. A bacteria which was CAMP test positive was considered as GBS. CAMP test culture results were re-tested using a Strp B Grouping Latex (Remel, USA).

GBS isolates were serotyped by using type-specific 10 antisera for serotypes Ia, Ib, II, III, IV, V, VI, VII, VIII, and IX (Statens Serum Institute, Denmark) as described by Slotved *et al*.^13^. GBS strain for serotyping was prepared first by growing on blood agar plates and then a heavy suspension of the test organism was prepared by using phosphate-buffered saline (PBS). A 20 µl aliquot of the bacterial suspension was placed on a disposable reaction card and mixed with 1 µl of latex suspension (reagents Ia, Ib, and II to IX; Strep-B-Latex kit; Statens Serum Institute, Copenhagen, Denmark). The card was rotated and observed for agglutination. A positive reaction was confirmed whenever agglutination appeared within the 30 s. All methods were performed in accordance with the relevant guidelines and regulations^1,2,12^

### Quality control

As part of quality in the study, we have included control strains such as *Streptococcus pyogenes* (ATCC 19615), *Streptococcus agalactiae* (ATCC12403), and *Staphylococcus aureus* (ATCC 24923*)*, during the study. The manufacture’s instruction was followed and negative and positive controls were during serotyping. To maintain the consistency, 5% of structured questionnaire were pretested at different hospital. To ensure the viability of GBS, all samples were placed in transport media immediately after collection and processed within four hours of collection.

### Statistical analysis

Data entry and analysis were done using computer with SPSS version 20 software. Prevalence figures were calculated for the total study population and separately by age group and risk factors. Logistic regression was used to a compare the results obtained from pregnant women and their newborns with different age groups, risk factors. A P-value less than 0.05 was considered significant.

### Ethics approval and consent to participate

The study was approved by the Institutional Review Board of College of Health Science, Addis Ababa University (Ref No: 069/13/DMIP) and the National Ethics and Research Committee (Ref No: 3.10/795/06). Written informed consent was obtained from all study participants. Written informed consent was also obtained from mothers to collect samples from their newborns. A guideline was followed as per the declaration of Helsinki for involving human participants in the study.

## Results

### Socio-demographic data

A total of 280 pregnant women along with their 282 newborns participated in this study. Majority of the participants were from Adama city 193(68.9%) followed by Lume 17(6.9%) and Fentale 11(3.9%). Most of the study participants were housewives 264(94.3%), followed by Nurses and Merchant. Two hundred one (71.8%) of the study participants were within the age range of 15–27 years. One hundred thirty seven (48.6%) newborns were males, and 145(51.4%) were females.

### Clinical and obstetric characteristics and GBS colonization rate of pregnant women

Among 280 pregnant women participated in the present study, 241(86.1%) delivered at gestational age of 37–42 (as measured by last menstrual period); 150(53.6%) were primigravida; 1(0.4%) had a history of newborns who developed EOD; 120(42.8%) had a previous history of vaginal delivery; 206(73.6%) had duration of rupture of membrane of 0–5 hours; 25 (9.3%) had premature rupture of membrane (PROM) (Table 1). Among 280 pregnant women, 37 of them were colonized with GBS giving maternal colonization rate of 13.2%, 95% CI [9.3–17.1]. GBS colonization rate among pregnant women at AHMC were not significantly associated with any of the risk factors (Table 1).

**Table 1:** Risk factors analysis for maternal GBS colonization, Adama Hospital Medical College (June 2014-October 2014) (n = 280).

Variables		Prevalence of GBS total GBS = 37	OR(95%CI)	P-value
Age group	15–27	27/201 (13.4%)	1.2 (0.54–2.69)	0.65
	>28	10/79 (12.7%)	1	
Gestational age	<37	2/27 (7.4%)	0.49 (0.11–2.2)	0.35
	37–42+	35/253 (13.8%)	1	
Gravida	Primigravida	21/150 (14%)	1.1 (0.54–2.2)	0.82
	Multigravida	16/130 (12.3%)	1	
Pervious mode of delivery	Vaginal	16/125 (12.8%)	0.9 (0.47–1.93)	0.8
	CS, Instrument & abortion	1/5(20%)	1.6 (0.17–15.2)	0.6
	NA	19/150 (12.6%)	1	
Duration of rupture of membrane	0–5 hr	28/206 (13.6%)	0.98 (0.32–3.03)	0.98
	6–10	5/45 (11.1%)	0.78 (0.19–3.19)	0.73
	11–15+	4/29 (13.8%)	1	
Premature rupture of membrane	Yes	4/26 (15.4%)	1.22 (0.39–3.76)	0.73
	No	33/254 (12.9%)	1	
Meconium stained amniotic fluid	Yes	4/57 (7.01%)	0.4 (0.15–1.28)	0.13
	No	33/223 (14.8%)	1	

CS = Cesarean Section NA = Not applicable OR = Odd ratio CI = Confidence interval GBS = Group B streptococcus.

### Characteristics of newborns and GBS colonization rate

Among 282 newborns participated in the present study, 269 (95.4%) were alive during birth; 246 (87.2%) were in weight range of 2500–4000 g; 179 (63.5%) had Appearance, Pulse, Grimace, Activity, Respiration (APGAR) score at 5 minutes >7; 247 (87.6%) had APGAR score at 10 minutes >7; 18 (6.4%) were newborns with other abnormalities (Table 2). From 282 newborns, 21 colonized with the GBS giving a newborns colonization rate of 7.4%, 95% CI [4.6–10.6]. All of 21 newborns colonized with GBS were from colonized mothers. Among 21 newborns colonized with GBS one developed signs and symptoms of EOD as confirmed by telephone interview. The symptoms were fever, repeated vomiting, irritability, bulged anterior fontanelle, abnormal breathing. From a total of 282 newborns, 13 (4.6%) were not alive during birth, among newborns who were not alive during birth one was colonized with GBS. None of the factor was significantly associated with GBS colonization (Table 2).

**Table 2:** Risk factors analysis for newborns GBS colonization, Adama Hospital Medical College (June 2014-October 2014) (n = 282).

Variables		Prevalence of total GBS = 21	OR(95%CI)	P-value
Sex of newborns	Male	10/137 (7.3%)	0.9 (0.39–2.30	0.92
	Female	11/145 (7.6%)	1	
Week of birth	<37	1/30 (3.3%)	0.4 (0.05–3.1)	0.38
	37–42+	20/252 (7.9%)	1	
APGAR score at 5 minutes	<7	5/103 (4.8%)	0.77 (0.26–2.44)	0.63
	>7	16/179 (8.9%)	1	
Status of new born during birth & immediate after birth	Dead	1/13 (7.7%)	1.03 (0.12-7-8.4)	0.97
	Alive	20/269 (7.4%)	1	
Developed EOD	Yes	1/4 (25%)	3.89 (0.38–38.7)	0.25
	No	20/278 (7.2%)	1	
Other abnormality	Yes	1/18 (5.6%)	0.78 (0.9–5.6)	0.75
	No	20/264 (7.6%)	1	

APGAR = Appearance, Pulse, Grimace, Activity, Respiration EOD = early onset disease.

### GBS serotype distribution

Out of 37 GBS isolates recovered from pregnant women the serotype distribution of Ia, Ib, II, III, V, VII, and NT were 6(16.2%), 8(21.6%), 10(27%), 3(8.1%), 8(21,6%), 1(2.7%), and 1(2.7%) respectively. Out of 21 GBS strained recovered from newborns the distribution of serotypes of Ia, Ib, II, III, V, VII were 3(14.3%), 6(28.6%), 6(28.6%), 4(19%), 1(4.8%), and 1(4.8%) respectively. All positive children have the same serotypes as their respective mothers (Table 3).

**Table 3:** Serotype distribution of GBS isolated from pregnant women and the newborns at Adama Hospital Medical College (June 2014-October 2014.

	GBS serotypes n (%)						
	Ia	Ib	II	III	V	VII	NT
Pregnant women, GBS = 37	6 (16.2%)	8 (21.6%)	10 (27%)	3 (8.1%)	8 (21.6%)	1 (2.7%)	1 (2.7%)
Newborn, GBS = 21	3 (14.3%)	6 (28.6%)	6 (28.6%)	4 (19%)	1 (4.8%)	1 (4.8%)	—
Pregnant women and newborns, n = 58	9 (15.5%)	14 (24.1%)	16 (27.6%)	7 (12.1%)	9 (15.5%)	2 (3.5%)	1 (1.7%)

NT = Non typeable, Ia, Ib, II, III, V, VII are serotypes of Group B streptococcus.

## Discussion

Maternal GBS colonization rate found in this study (13.2%) was comparable with maternal GBS colonization rate reported from Nekemte, Ethiopia (12.2%)^14^, Addis Ababa, Ethiopia (14.6%)^15^, Eastern Ethiopia (13.68%)^16^, Namibia (13.6%)^17^, Kenya (12%)^18^, Western Cape, South Africa (16.6%)^19^. Previous study conducted in Hawassa showed higher maternal GBS colonization rate than this study^11,20^. Our finding is low compared to GBS colonization rate reported from Congo (20%)^21^, Malawi (21.7%)^22^, Greter Acra (26.8)^23^, Uganda (28.8%)^24^, South Africa (28.4%)^25^, Zimbabwe (31.6%)^26^, South Africa (37%)^17^, Egypt (26.25%)^27^, Tanzania (23%)^28^, Jordan (19.5%)^29^, and Gabon (19%)^30^. However, it was high compared to Nigeria (8.3%)^31^ and Sudan (0.5%)^32^. Maternal GBS colonization rate found in the present study was comparable to maternal GBS colonization rate reported from Brazil (14.6%)^33^ Thai-Myanmar (12%)^34^, however, it is low compared to Germany (16%)^35^, USA (21%)^36^, Sweden (25.4%)^37^, The Netherlands (21%)^38^, and New Zealand 20%^39^.

There are several reasons for the variation of maternal GBS colonization rates in different countries and within the countries. The variation can be a true difference or it can be due to laboratory methods used, time and site of sample collection^25^. Use of selective media, such as limborth instead of direct plating on blood agar increase the positivity rate. Taking sample both from vagina and rectal area increase the positivity rate as compared to swabbing only from the vagina^1^.

About 50% of newborns from GBS colonized mothers are GBS positive during birth. Most newborns who acquire GBS during passage through the birth canal remain healthy. About 1–3% of them may develop invasive disease^3^. In the present study, 7.4% of newborns were colonized with GBS, which is in line with earlier studies conducted in industrialized countries^40,41^. Out of 21 GBS colonized newborns in the present study one of them developed signs and symptoms of EOD as followed by telephone interviews. In this study, out of 13 newborns who were not alive during birth one of them was colonized with GBS. Since other studies are also linking stillbirth with GBS, this area needs further investigation^4^.

Group B streptococcus colonization rate varies according to region, ethnicity, socioeconomic status, maternal age, gestational age less than 37, and prolonged rupture of the membrane^7^. In this study no significant association between GBS colonization rate and the measured risk factors were found (P > 0.05).

Capsular polysaccharide-based serotyping has been applied for epidemiological studies of GBS and it is important in the development of multivalent vaccines containing several serotypes^32,42^. GBS serotype distribution varies geographically and over time^43^. In European countries, The United States and in South America serotypes Ia, Ib, III or V are the most frequently reported GBS serotypes. From Japan serotype VI and VII are frequently reported^7,43^. The invasiveness of GBS also varies according to GBS serotypes; serotype III is believed to be the most virulent GBS serotypes^43^.

In the present study, the prevalent serotype was serotype II (27.6%) followed by serotype Ib (24.1%), Ia (15.5%), and V (15.5%). The finding of this study was in partial agreement with GBS serotypes reported from other countries^44,45^. The prevalence of serotype III in our study is low compared to the report from many other countries^10,43^. A study from S. Africa reported high serotype Ia (39.2%), III (32.8%) compared to our study^25^, however, they have reported comparable prevalence of serotype V (12.4%)^25^. The prevalence of serotype Ia in this study was comparable to a study conducted in Zimbabwe (17%)^26^, but they have reported a high prevalence of serotype III (47.7%) and V (23.2%) than our finding^26^. Compared to our study, a low prevalence of serotype II (6.4%) and high prevalence of serotype V (30.3%), III (27.5%) was reported from Gabon. The prevalence of serotype Ia (12.8%), Ib (22.9%) is comparable with our study^26^.

Edmonds *et al*. (2012) in their review, reported that serotype III to be the most common (48.9%) among different geographic areas including in Africa, followed by serotypes Ia, Ib, II, and V. A trivalent (Ia, Ib, and III) the conjugate vaccine developed by Novartis was shown to be effective. However, the distribution of serotype is not uniform in different countries and it will change over time. To solve this problem, Pfizer, in 2017, started to evaluate a Pentavalent GBS polysaccharide capsular vaccine containing GBS serotype Ia, Ib, II, III, and V^25,46–48^. Both trivalent and Pentavalent vaccine does not contain all GBS serotypes: IV, VI, VII, VIII, and IX are missed from the formulation. This indicates data on GBS serotype distribution from each country is highly required for appropriate vaccine formulation.

As we have used convenience sampling technique the finding of this study may not represent all pregnant mothers in the study area. In this study we did not used all known serotypes of GBS for quality control as they are not available locally.

## Conclusion

Group B streptococci have been recognized as the leading cause of infectious meningitis in infants in high-income countries but its importance in developing countries is not clear. The primary risk factor for EOD due to the GBS is GBS colonization of pregnant women before delivery. Maternal and newborns GBS colonization rate and GBS serotype distribution in this study is similar to other countries. The most prevalent GBS serotypes detected in our study include; serotype II, Ib, Ia, V, and III. Vaccine covering these five serotypes may prevent most of EOD due to GBS in our study area if the disease-causing GBS serotypes is similar to colonizing GBS serotypes. Based on this study we recommend large scale surveillance of neonatal disease due to GBS, circulating serotypes and adoption of prevention strategy.

## Data Availability

All relevant data are available within the paper.
